# The frequency of blindness and visual impairment in the central-west region of Brazil

**DOI:** 10.5935/0004-2749.202100104

**Published:** 2021

**Authors:** Guilherme B. Ribeiro, Giuliano P. Dobri, Ricardo Y. Abe

**Affiliations:** 1 Hospital Oftalmológico de Brasília, Brasília, DF, Brazil

Dear Editor,

It is estimated that approximately 1.1 million people worldwide experience vision loss primarily because they do not have access to eye care services. Over 90% of those people live in lowand middle-income countries, 74% are older than 50 years, and 55% are women^(1)^. The World Health Organization defines blindness as best-corrected visual acuity (BCVA) of worse than 1.3 logMAR in the better eye and low vision or visual impairment as best-corrected visual acuity of 0.5 to 1.3 logMAR in the better eye^(2)^.

According to the most recent survey by the Brazilian Geography and Statistic Institute, 3.6% of the Brazilian population (7.2 million citizens) have blindness or visual impairment^(3)^. Nonetheless, data regarding blindness and low vision frequencies in the central-west region of Brazil are scarce. We therefore investigated the frequency and causes of visual impairment and blindness in a reference visual care foundation in Brasília during the years 2016 to 2018.

A total of 3002 medical records from first appointments in the clinic were analyzed. Of these, 258 cases of blindness and visual impairment were eligible for this study; 101 (39.1%) of the patients were men, and 157 (60.9%) were women. The mean age of the patients was 67.77 ± 16.85, and their ages ranged from 6 to 98 years. The mean BCVA in the first appointment was 0.97 ± 0.38 logMAR.

To analyze the causes of visual impairment and blindness, we divided the 258 patients into two groups: 86 patients who had the same BCVA in both eyes and 172 patients who had different BCVAs. The most frequent primary causes in the latter group were cataract (113 cases [65.7%]), glaucoma (11 cases [6.4%]), and posterior capsule opacification (10 cases [5.8%]; Table 1). Of those 172 patients, 97 received ocular treatment after the first clinic appointment. The mean BCVAs before and after treatment were 0.90 ± 0.33 logMAR and 0.26 ± 0.39 logMAR, respectively (p<0.001, *t* test). After the treatment, 86 patients (88.7%) experienced improvement in their vision by 0.5 logMAR or better; of the other 11 patients (11.3%); however, 7 (7.2%) experienced no improvement in BCVA after the procedure, and the others experienced worsening of BCVA by more than 0.5 logMAR. For all 11, glaucoma was the primary cause of visual impairment.

**Table 1:** Most prevalent causes of visual impairment and blindness among 172 patients

Etiology	n
Cataract	113 (65.7%)
Glaucoma	11 (6.4%)
Posterior capsule opacification	10 (5.8%)
Keratoconus	8 (4.7%)
Diabetic retinopathy	8 (4.7%)

Among the 86 patients who had the same BCVA in both eyes, the most frequent causes of visual impairment in the right eyes were cataract (64 cases [74.4%]), glaucoma (5 cases [5.8%]) and posterior capsule opacification (7 cases [5.8%]). Among the left eyes, the most frequent causes of visual impairment were cataract (62 cases [72.0%]), glaucoma (5 cases [5.8%]), and posterior capsule opacification (5 cases [5.8%]; Table 2).

**Table 2:** Most prevalent causes of visual impairment and blindness in 86 patients with the same best-corrected visual acuity in both eyes

Etiology	n	
	Right eyes	Left eyes
Cataract	64 (74.4.7%)	62 (72.0%)
Glaucoma	5 (5.8%)	5 (5.8.2%)
Posterior capsule opacification	7 (5.8%)	5 (5.8.2%)
Keratoconus	2 (2.3%)	2 (2.3%)
Diabetic retinopathy	2 (2.3%)	2 (2.3%)

According to our findings, cataract was the leading cause of visual impairment, which corroborates the findings reported in global populations^(4)^. Of our patients, 89% were older than 50 years, and 57.91% were women. Similar results were found in the Los Angeles Latino Eye Study, in which 80% of visually impaired people worldwide were 50 years of age or older. That study also demonstrated that visual impairment increased with age and was more severe in women^(5)^.

Despite being the most prevalente cause of visual impairment, the visual loss from cataract is reversible. The high frequency of irreversible visual impairment and blindness in patients with glaucoma is of great concern. Of the 97 patients who received ocular treatment in our sample, the 7 patients (7.2%) who did not experience improvement in BCVA after treatment had glaucoma. The Brazilian population is growing in number and is aging; thus the risk of blindness and visual impairment might increase further. Our findings help clarify the frequency of blindness and visual impairment in the central-west region of Brazil and highlight the importance of access to proper eye care in minimizing the risk of blindness and visual impairment.
